# Oxidation-enhanced thermoelectric efficiency in a two-dimensional phosphorene oxide

**DOI:** 10.1038/s41598-021-97943-8

**Published:** 2021-09-17

**Authors:** Seungjun Lee, Jeong-Pil Song, Seoung-Hun Kang, Young-Kyun Kwon

**Affiliations:** 1 Department of Physics, Kyung Hee University, Seoul, 02447 Korea; 2 Department of Physics, University of Arizona, Tucson, AZ 85721 USA; 3 Materials Science and Technology Division, Oak Ridge National Laboratory, Oak Ridge, TN 37831 USA; 4 Department of Information Display and Research Institute for Basic Sciences, Kyung Hee University, Seoul, 02447 Korea

**Keywords:** Electronic properties and materials, Thermoelectrics, Two-dimensional materials

## Abstract

We performed density functional theory calculations to investigate the thermoelectric properties of phosphorene oxide (PO) expected to form by spontaneous oxidation of phosphorene. Since thermoelectric features by nature arise from the consequences of the electron-phonon interaction, we computed the phonon-mediated electron relaxation time, which was fed into the semiclassical Boltzmann transport equation to be solved for various thermoelectric-related quantities. It was found that PO exhibits superior thermoelectric performance compared with its pristine counterpart, which has been proposed to be a candidate for the use of future thermoelectric applications. We revealed that spontaneous oxidation of phosphorene leads to a significant enhancement in the thermoelectric properties of *n*-doped phosphorene oxide, which is attributed to the considerable reduction of lattice thermal conductivity albeit a small decrease in electrical conductivity. Our results suggest that controlling oxidation may be utilized to improve thermoelectric performance in nanostructures, and PO can be a promising candidate for low-dimensional thermoelectric devices.

## Introduction

Over the last few decades, much attention has been paid to some particular materials that efficiently convert heat into electricity or vice versa for their potential applications in thermoelectric power generating or cooling systems^1,2^. Their thermoelectric efficiencies can be assessed at temperature *T* by a dimensionless figure of merit

1 $$\begin{aligned} ZT=\frac{S^2\sigma {T}}{\kappa }, \end{aligned}$$

where *S*, $\sigma$, and $\kappa$ are Seebeck coefficient, electrical and thermal conductivities. The thermal conductivity can be further decomposed into the electronic and lattice thermal conductivities $\kappa _e$ and $\kappa _l$, or $\kappa =\kappa _e+\kappa _l$. A promising thermoelectric candidate with a large *ZT* value should concurrently possess *incompatible* characteristics: high electrical conduction with a high $\sigma$ and efficient thermal insulation with a low $\kappa$, not to mention high Seebeck coefficient, which is usually observed in semiconductors. Intriguingly, such a conflicting condition has recently been realized in some chalcogenides such as bismuth telluride ($\hbox {Bi}_2\hbox {Te}_3$)^1^, antimony telluride ($\hbox {Sb}_2\hbox {Te}_3$)^3^, tin selenide (SnSe)^4,5^, and copper selenide ($\hbox {Cu}_2\hbox {Se}$)^6,7^. Although some of these materials are currently being used in thermoelectric devices, their toxicity and/or expensive materials cost will hinder future thermoelectric applications^8^.

As alternatives that would provide inexpensive and environmentally-friendly thermoelectricity, two-dimensional (2D) materials have drawn much attention since, in general, they tend to have high values of Seebeck coefficient originating from the effect of the low-dimensionality, such as an abrupt change in the density of states (DOS)^9^ and a quantum confinement effect^10,11^. Nevertheless, it turned out that some 2D materials failed to satisfy such expectations. For instance, the Seebeck coefficient of graphene is small because of its metallic nature^12^, and $\hbox {MoS}_2$ exhibited a low *ZT* value around 0.05 attributed to its relatively large thermal conductivity^13^.

After the discovery of a single layer of black phosphorus or phosphorene^14^, there have been several theoretical studies proposing that phosphorene would be a candidate material for thermoelectric applications^15–18^. Based on numerical calculations performed within the constant relaxation time approximation using the deformation potential theory, they reported that phosphorene, which is environmentally friendly or non-toxic, has not only a large Seebeck coefficient originating from its semiconductivity, but also highly anisotropic transport behaviors of high electrical and low thermal conductivities along a certain direction caused by its intrinsic puckered structure^15,16^. An explicit inclusion of electron-phonon interactions, however, revealed that those reported room-temperature *ZT* values were an order of magnitude overestimated^19^, implying that phosphorene may not be a suitable choice for thermoelectric applications. To make it worse, phosphorene is known to be structurally unstable under the atmospheric conditions and thus can be easily oxidized due to the lone pair electrons of each phosphorus atom^20–24^.

Here we propose that spontaneous oxidation of phosphorene may be a resolution of the issues in phosphorene for a thermoelectric purpose. To correctly describe the thermoelectric properties of both phosphorene and PO or to calculate their *ZT* values accurately, we explicitly evaluated the electron relaxation times $\tau _{n,{\mathbf {k}}}$ for given electronic band indices *n* and electron wave vectors ${\mathbf {k}}$ in the presence of electron-phonon interactions. Then a set of $\{\tau _{n,{\mathbf {k}}}\}$ was used to solve the Boltzmann transport equation based on the first-principles density functional theory to obtain the electronic contributions to the quantities in Eq. (1). Combining them with their lattice thermal conductivity calculated in our earlier study^24^, we determined their thermoelectric-related characteristics. It was found that the *p*-type phosphorene exhibits better thermoelectricity along the armchair direction but worse along the zigzag direction than PO, which shows a much smaller directional dependence. Furthermore, we found that the *n*-type PO is thermoelectrically superior not only to the *n*-type phosphorene regardless of their directions but also to the *p*-type phosphorene. This result is attributed to a significant reduction in $\kappa _l$ originating from spontaneous oxidation^24^ albeit a small decrease in power factor $S^2\sigma$. *ZT* values of PO were estimated to range from 0.1 to 0.5 at $T=300$ to 700 K in the electron doping concentration. Therefore, *n*-doped PO would be a promising and eco-friendly thermoelectric material, and controlling the degree of oxidation may be a useful strategy to improve the thermoelectric properties of the material.

## Results and discussion

**Figure 1:**
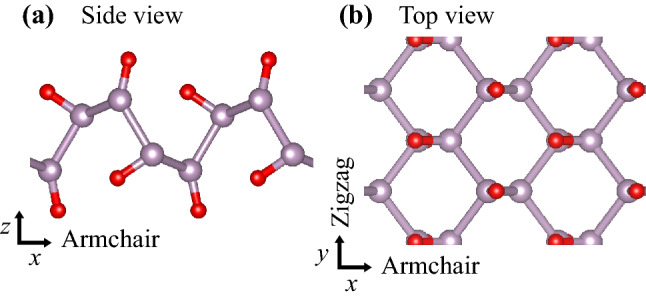
(**a**) Side and (**b**) top view of the equilibrium structure of PO. Phosphorus and oxygen atoms are depicted by purple and red spheres, respectively. For convenience, the armchair and zigzag directions were chosen as the *x*- and *y*-axes, and the *z*-axis was selected to be the out-of-plane direction.

Various oxidation processes of phosphorene were reported earlier^20–24^. Our previous studies^23,24^ showed that fully-oxidized phosphorene or PO possesses highly-anisotopic configuration similar to its pristine counterpart, as shown in Fig. 1. We revealed that PO with a direct bandgap of 0.83 eV possesses two nonsymmorphic symmetries with the inversion symmetry broken, guaranteeing symmetry-protected band structures including the band degeneracy and four-fold degenerate Dirac points^23^. It was also shown that the flexible bonds between phosphorus and oxygen atoms in PO lead to a significant decrease in the lattice thermal conductivity $\kappa _l^{\mathrm {PO}}$, which was evaluated to be in the range between 2.4 and 7.1 W/mK depending on the transport direction, compared to that of pristine phosphorene $\kappa _l^{\mathrm {P}}$ in the range of 65 and 146 W/mK^24^. This reduction implies that PO would provide a favorable condition for thermoelectric performance.

**Figure 2:**
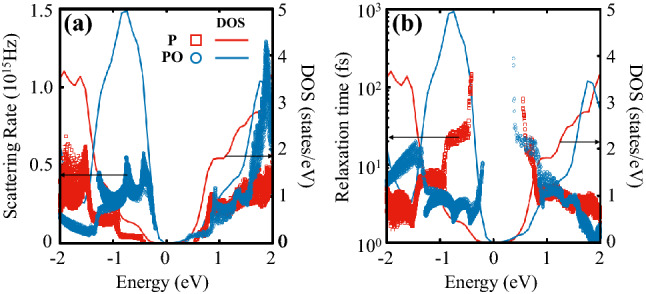
(**a**) Electron-phonon scattering rates and (**b**) electron relaxation times $\tau _{n,{\mathbf {k}}}(E)$ of phosphorene (P), marked with red $\Box$ symbols, and phosphorene oxide (PO) with blue﻿ $\bigcirc$ symbols, as a function of energy. Each data point corresponds to an available initial state $|n,{\mathbf {k}}\rangle$ at $T=300$ K. For comparison, their corresponding total densities of states are overlaid by the solid lines with the same colors with respect to the auxiliary *y* axis. Note that the relaxation times in (**b**) are given in a logarithm scale.

To consider the effect of the electron–phonon interaction on transport properties such as $\sigma$ and $\kappa$, we evaluated the electron–phonon scattering rate and the carrier relaxation time, which is the inverse of the former, of both phosphorene and PO for the all available initial states $|n,{\mathbf {k}}\rangle$. According to the Fermi’s golden rule, the electron scattering rate is proportional to the number of available final states satisfying conservation conditions. As seen in Fig. 2a, the electron-phonon scattering rates are somewhat roughly proportional to their corresponding total DOSs, implying the modification of DOS near band edges would be a viable strategy for enhancing the transport properties. We further observed that the carrier relaxation time of PO is larger near the lowest conduction band but much smaller near the highest valance band than that of phosphorene, as shown in Fig. 2b. This indicates that *n*-type PO would be a better choice for a thermoelectric material than *p*-type PO, because the carrier relaxation time is closely associated with the spectral conductivity tensor $\varvec{\Sigma }$ as described in Eq. (3), and thus with the conductivity $\varvec{\sigma }$.

**Figure 3:**
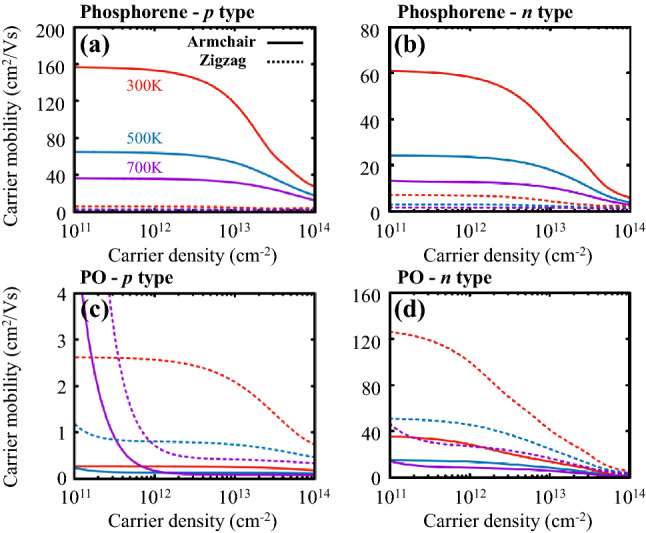
Carrier mobilities for (**a**) *p*- and (**b**) *n*-type phosphorene, and (**c**, **d**) their PO counterparts as a function of 2D carrier density. Solid and dashed lines denote mobilities computed along the armchair and zigzag transport directions at three different temperatures of 300, 500, and 700 K represented with the red, blue, and purple colors, respectively.

We used the calculated relaxation time to evaluate the carrier mobility tensor $\varvec{\mu }$ of phosphorene and PO as a function of carrier density concentration at various temperatures, by dividing the electrical conductivity tensor $\varvec{\sigma }$ by carrier density. Figure 3 shows the evaluated carrier mobility, $\mu _{\mathrm {M,X}}^a$, of phosphorene (M = P) and PO (M = PO) as a function of the carrier, either hole ($a$ = $p$) or electron ($a$ = $n$), density ($\rho _p$ or $\rho _n$) along the armchair (X = A) and zigzag (X = Z) direction at three different temperature values. As expected, the carrier mobility clearly exhibits anisotropic behaviors for both phosphorene and PO due to their anisotropic puckered geometries. For phosphorene, we observed that both hole (*p*-type) and electron (*n*-type) mobilities, $\mu ^p_{\mathrm {P,Z}}$ and $\mu ^n_{\mathrm {P,Z}}$, are diminutive along the zigzag direction, independent of the type of carrier, whereas those along the armchair direction $\mu ^p_{\mathrm {P,A}}$ and $\mu ^n_{\mathrm {P,A}}$ are substantially high, as shown in Fig. 3a and b. For instance, we evaluated $\mu ^p_{\mathrm {P,A}}\approx 150$ $\hbox {cm}^{2}$/Vs and $\mu ^n_{\mathrm {P,A}}\approx 60$ $\hbox {cm}^{2}$/Vs for their respective carrier density values of $1 \times 10$ $^{12}$ $\hbox {cm}^{-2}$ at room temperature (*T* = 300 K). These results are in good agreement with previously evaluated values^19,25^. For PO, on the other hand, the hole mobility $\mu ^p_{\mathrm {PO}}$ is quite low varying, from 0.25 to 2.6 $\hbox {cm}^2$/Vs, as shown in Fig. 3c, due to short carrier relaxation time near the valence band edge compared to that of phosphorene presented in Fig. 2b, whereas the electron mobility exhibits much higher values, for instance, $\mu ^n_{\mathrm {PO,Z}}\approx 129$ $\hbox {cm}^{2}$/Vs with $\rho _n\approx 10^{11}$ $\hbox {cm}^{-2}$ at $T=300$ K, as shown in Fig. 3d originating from a significant reduction in the electron-phonon scattering. Note that, in any cases, the mobility tends to decrease as temperature increases because of the enhancement of electron scattering.

**Figure 4:**
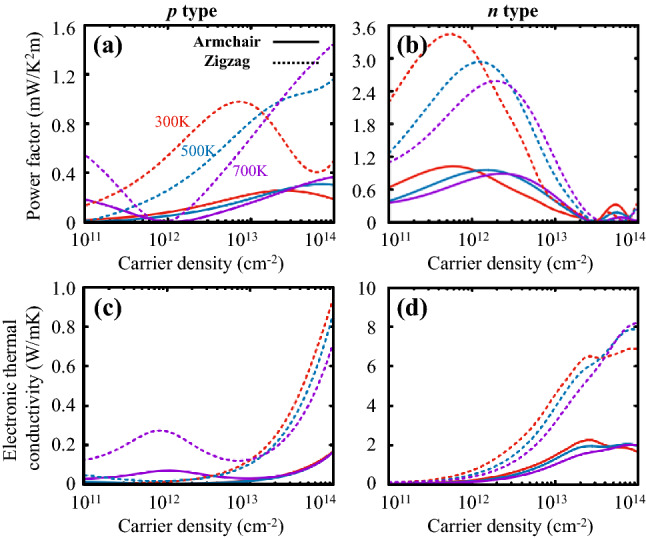
Power factors for (**a**) *p*- and (**b**) *n*-type PO, and (**c**, **d**) their respective electronic thermal conductivities as a function of 2D carrier density. Solid and dashed lines denote these quantities computed along the armchair and zigzag transport directions at three different temperatures of 300, 500, and 700 K represented with the red, blue, and purple colors, respectively.

The relaxation time was also fed into Eq. (3) to evaluate the spectral conductivity tensor $\varvec{\Sigma }(E,T)$ and thus the moments $\varvec{\mathscr {L}}^{(\alpha )}(\mu ,T)$ for various transport coefficients given in Eq. (4). Such moments were employed to calculate the electric conductivity, the Seebeck coefficient, and the electronic thermal conductivity tensors using Eqs. (5) – (7). The components corresponding to a specific transport direction, either the armchair or zigzag direction, were extracted from the transport tensors to assess the thermoelectric figure of merit *ZT* given in Eq. (1) along the selected transport direction. Figure 4 shows the computed power factors ($S^2\sigma$) and the electronic thermal conductivities ($\kappa _e$) of both *p*- and *n*-type PO as a function of the carrier density at three different temperatures of 300, 500, and 700 K. Both $S^2\sigma$ and $\kappa _e$ are not only highly anisotropic similar to the mobility presented in Fig. 3, but they also depend strongly on the type of carriers. Particularly, the power factors evaluated at $T=300~\hbox {K}$ have a peak at $\rho _p\approx 10^{13}\hbox {cm}^{-2}$ and $\rho _n\approx 10^{12}\hbox {cm}^{-2}$ for the *p*- and *n*-type PO, respectively. These carrier density values can be experimentally feasible since similar values were already achieved in other 2D thermoelectric materials, such as graphene^26^ and transition metal dichalcogenides^27,28^. For *n*-type PO, the maximum of the power factor at $T=300~\hbox {K}$ is about $3.4~\hbox {mW}/\hbox {K}^2\hbox {m}$, which is more than three times higher than that for the *p*-type one, as shown in Fig. 4a and b. It is noted that this value is still smaller than values of $\sim 29~\hbox {mW}/\hbox {K}^2\hbox {m}$ and $\sim 6.5~\hbox {mW}/\hbox {K}^2\hbox {m}$ for *p*- and *n*-type phosphorene, respectively, evaluated at $T = 300~\hbox {K}$, as shown in Fig. S1a and b in Supplementary Information. For the *n*-doped case, however, since the reduction in the lattice thermal conductivity by $\sim 1/26$ times^24^ is much more dominant than that in power factor, the *n*-doped PO may still have high *ZT* values in spite of such low power factors.

In most semiconductors, the electronic contribution to the thermal conductivity $\kappa _e$ is usually much smaller than the lattice contribution $\kappa _l$, and thus the former may provide a negligible effect on the thermoelectric efficiency or *ZT*. For example, phosphorene has $\kappa _e <10~\hbox {W/mK}$ at $T=300~\hbox {K}$ with a moderate doping concentration range from $1\times 10^{12}$ to $1\times 10^{13}~\hbox {cm}^{-2}$, which is much smaller than its lattice thermal conductivity^24^, as shown in Fig. S1c and d in Supplementary Information. $\kappa _e$ of PO, however, plays a crucial role in determining its *ZT* value, due to a significant reduction of $\kappa _l$ in PO^24^. Figure 4c and d shows the evaluated $\kappa _e$ of PO as a function of the carrier density of holes and electrons. Similar to its mobility and power factor, $\kappa _e$ of PO has higher values along the zigzag direction than along the armchair direction, which satisfies the Wiedemann-Franz law. Especially for electron-doped cases, the electronic thermal conductivity $\kappa _e$ of *n*-type PO reached $\sim 4~\hbox {W/mK}$, which is comparable to the $\kappa _l^{PO}$, at around a carrier density of $\sim 10^{13}~\hbox {cm}^{-2}$. More interestingly, $\kappa _e$ becomes even greater than $\kappa _l$ at higher temperatures because $\kappa _l$ is inversely proportional to *T*^24^. It is, thus, inevitable to consider the electronic contribution to the thermal conductivity for an accurate estimation of the *ZT* value of PO.

**Figure 5:**
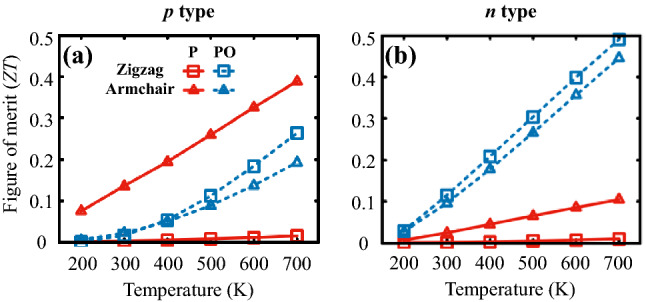
Thermoelectric figures of merit (*ZT*) of (**a**) *p*- and (**b**) *n*-type phosphorene (red solid lines) and PO (blue dashed lines) as a function of temperature. Square and triangle symbols denote *ZT* computed along the zigzag and armchair transport directions. We used $\rho _p=6.4\times 10^{12}~\hbox {cm}^{-2}$ and $\rho _n=4.8\times 10^{12}~\hbox {cm}^{-2}$ for phosphorene and $\rho _p=1.0\times 10^{14}~\hbox {cm}^{-2}$ and $\rho _n=1.2\times 10^{12}~\hbox {cm}^{-2}$ for PO, with which the respective figures of merit was estimated to reach their corresponding maxima at $T=700~\hbox {K}$, as shown in Fig. S2 in Supplementary Information.

With all these computed quantities, we are now ready to evaluate the thermoelectric figures of merit *ZT* of PO and phosphorene for comparison. Figure 5 summaries the temperature dependence of *ZT* of *p*- and *n*-type phosphorene and PO along the zigzag and armchair transport directions computed with each particular value of hole and electron carrier density, with which their *ZT* values reached to its maximum at $T=700$ K as shown in Fig. S2 in Supplementary Information. The *p*-type phosphorene exhibits a better thermoelectric performance along the armchair direction than not only along the zigzag direction but also along any directions for the *n*-type counterpart, due mainly to its higher power factors shown in Fig. S1 in Supplementary Information. Its *ZT* value reaches $\sim$0.4 at 700 K along the armchair direction in spite of its large $\kappa _l$. On the other hand, PO shows better thermoelectric performance in the electron-doped case than in the hole-doped case. Particularly, the $ZT\approx 0.5$ evaluated at 700 K along the zigzag direction is significantly larger than $ZT\approx 0.03$ of phosphorene. It interestingly turned out that the thermoelectric efficiency in the electron doped PO becomes almost isotropic with the *ZT* value of $\sim 0.45$ along the armchair direction. It is suggested that the oxidation in phosphorene diminishes thermoelectric anisotropy observed in pristine phosphorene. This diminution in anisotropy is explained by the opposite directional trends in the power factor and lattice thermal conductivity^24^. Furthermore, the optimal electron doping concentration ($\rho _n=1.2\times 10^{12}$ $\hbox {cm}^{-2}$) for PO is much smaller than that ($\rho _p=6.4\times 10^{12}~\hbox {cm}^{-2}, \rho _n=4.8\times 10^{12}$ $\hbox {cm}^{-2}$) for phosphorene. This is a very good advantage because high concentration doping is not only hardly achievable in reality but also in heavily doped semiconductors; there would be significant impurity scattering effects, which are difficult to be treated directly in theoretical calculations.

## Summary and conclusions

We presented the thermoelectric properties of a new 2D material, phosphorene oxide, investigated by performing first-principles calculations. For a precise evaluation of its thermoelectric efficiency, we accurately calculated phonon-mediated electrical conductivity and lattice thermal conductivity with anharmonic phonon effects. We found that the highly anisotropic structure of the PO results in anisotropic electrical and thermal transport properties. Nevertheless the thermoelectric efficiency of the *n*-doped PO is almost isotropic or independent of the transport directions. Our results suggest that the *n*-type PO is superior in the thermoelectric behaviors to the *p*-type phosphorene due mainly to a significant reduction in lattice thermal conductivity albeit a small decrease in power factor. It is worth noting that spontaneous oxidation of phosphorene improves overall thermoelectric properties as well as structural stability, and thus the *n*-type PO would be a potential candidate for environmentally-friendly and cost-effective thermoelectric applications.

## Methods

To investigate thermoelectric properties of phosphorene and PO, we first calculated the electronic structures by carrying out first-principles calculations based on the density functional theory^29^ using Quantum Espresso code^30^. Plane wave basis was used to expand the electronic wavefunctions with a kinetic energy cutoff of 80 Ry. The core and valence electrons were represented by the norm-conserving pseudopotentials^31,32^, and the exchange-correlation functional was treated within the generalized gradient approximation of Perdew–Burke–Ernzerhof^33^. To avoid interactions from layers in neighboring cells, we added a vacuum slab of 20 Å parallel to the layer in the unit cell. The Brillouin zone (BZ) was sampled using a $\Gamma$-centered $10\times 10\times 1$ *k*-points mesh for the integration over the BZ. Then, the phonon dispersion was calculated using the density functional perturbation theory (DFPT)^34^ with a $10\times 10\times 1$ *q*-points grid.

As a next step, we explicitly evaluated the energy- and temperature-dependent relaxation time $\tau _{n,{\mathbf {k}}}(E,T)$ for given *n* and ${\mathbf {k}}$ defined as^35,36^

2 $$\begin{aligned} \begin{aligned} \frac{1}{\tau _{n,{\mathbf {k}}}(E,T)}&= 2\pi \sum _{m,\nu } \int \frac{d{\mathbf {q}}}{\Omega _{\mathrm {BZ}}} |\mathscr {G}_{mn,\nu }({\mathbf {k}},{\mathbf {q}})|^2 \\&\quad \times \left\{ \left[ f(E_{m,{\mathbf {k}}+{\mathbf {q}}},T) +g(\omega _{\nu ,{\mathbf {q}}},T)\right] \delta (E-E_{m,{\mathbf {k}} +{\mathbf {q}}}-\omega _{\nu ,{\mathbf {q}}})\right. \\&\quad \times \left. \left[ 1-f(E_{m,{\mathbf {k}}+{\mathbf {q}}},T) +g(\omega _{\nu ,{\mathbf {q}}},T)\right] \delta (E-E_{m,{\mathbf {k}} +{\mathbf {q}}}+\omega _{\nu ,{\mathbf {q}}})\right\} , \end{aligned} \end{aligned}$$

in atomic units ($\hbar =e=1$). Here $\sum _{m,\nu }$ and $\int {d{\mathbf {q}}/\Omega _{\mathrm {BZ}}}$ represent respectively the summation over both electronic band and phonon branch indices *m* and $\nu$, and the normalized ${\mathbf {q}}$-space integration over the whole BZ with its volume $\Omega _{\mathrm {BZ}}$. The Fermi–Dirac and Bose–Einstein distributions, $f(E_{m,{\mathbf {k}}+{\mathbf {q}}},T)$ and $g(\omega _{\nu ,{\mathbf {q}}},T)$ represent the electronic and phononic occupations for their corresponding energy eigenvalues $E_{m,{\mathbf {k}}+{\mathbf {q}}}$ and $\omega _{\nu ,{\mathbf {q}}}$ at temperature *T*. $\delta (\epsilon )$ is the usual Dirac delta function. $\mathscr {G}_{mn,\nu }({\mathbf {k}},{\mathbf {q}})$ is the first-order electron–phonon matrix element given by $\langle m,{\mathbf {k}}+{\mathbf {q}}|\partial _{\nu ,{\mathbf {q}}}V|n,{\mathbf {k}} \rangle /\sqrt{2\omega _{\nu ,{\mathbf {q}}}}$ with $\partial _{\nu ,{\mathbf {q}}}V$ the derivative of the self-consistent potential associated with a phonon mode $\omega _{\nu ,{\mathbf {q}}}$^34,37^. It is related to the probability amplitude of phonon-mediated scattering process from a state $|n,{\mathbf {k}}\rangle$ to another $|m,{\mathbf {k}}+{\mathbf {q}}\rangle$. $\mathscr {G}_{mn,\nu }({\mathbf {k}},{\mathbf {q}})$ was computed by employing DFPT on $10 \times 10$ $\times$1 coarse *k*- and *q*-points meshes and then using maximally localized Wannier functions^38–40^ through the EPW package^35^ to interpolate over $100 \times 100$ $\times$1 fine *k*- and *q*-points grids.

For various transport-related quantities, we solved the semi-classical Boltzmann transport equation^41,42^. The computed electron relaxation times $\tau _{n,{\mathbf {k}}}$ given in Eq. (2) together with the group velocity ${\mathbf {v}}_{n,{\mathbf {k}}}=\nabla _{\mathbf {k}}E_{n,{\mathbf {k}}}$ calculated from the electronic structure were used to computed the spectral conductivity tensor given as

3 $$\begin{aligned} \varvec{\Sigma }(E,T)=\frac{1}{\Omega }\sum _{n}\int \frac{d{\mathbf {k}}}{\Omega _{\mathrm {BZ}}}{\mathbf {v}}_{n,{\mathbf {k}}} \otimes {\mathbf {v}}_{n,{\mathbf {k}}}\tau _{n,{\mathbf {k}}}(E,T)\delta (E-E_{n,{\mathbf {k}}}), \end{aligned}$$

where $\Omega$ is the unit cell volume, and the symbol $\otimes$ denotes the standard outer product. Since phosphorene and PO are 2D systems, the evaluation of the conductivity tensor requires the thicknesses of the systems, which were set to 5.5 and 8.0 Å, respectively, corresponding approximately to the half of the out-of-plane lattice constants of their bulk configurations. The computed spectral conductivity tensor was used to evaluate the moments with an order $\alpha$ $(\alpha =0,1,2,\cdots )$ of the generalized transport coefficients as a function of chemical potential $\mu$ and temperature *T* defined by

4 $$\begin{aligned} \varvec{\mathscr {L}}^{(\alpha )}(\mu ,T)=\int {dE\varvec{\Sigma } (E,T)(E-\mu )^\alpha \left[ -\frac{\partial {f(E-\mu ,T)}}{\partial {E}}\right] }. \end{aligned}$$

These moments are directly related to various transport coefficients, such as electrical conductivity, Seebeck coefficient, and electronic thermal conductivity tensors, as

5 $$\begin{aligned} \varvec{\sigma }(\mu ,T)&=\varvec{\mathscr {L}}^{(0)} \end{aligned}$$

6 $$\begin{aligned} {\varvec{S}}(\mu ,T)&=\frac{1}{T}\varvec{\mathscr {L}}^{(1)} \otimes \frac{1}{\varvec{\mathscr {L}}^{(0)}} \end{aligned}$$

7 $$\begin{aligned} \varvec{\kappa _e}(\mu ;T)&=\frac{1}{T}\left[ \varvec{\mathscr {L}}^{(2)} -\left( \varvec{\mathscr {L}}^{(1)}\otimes \varvec{\mathscr {L}}^{(1)}\right) \otimes \frac{1}{\varvec{\mathscr {L}}^{(0)}}\right] , \end{aligned}$$

where $1/\varvec{\mathscr {L}}^{(\alpha )}$ represents the element wise inversion of $\varvec{\mathscr {L}}^{(\alpha )}$.

## Supplementary Information

Supplementary Information.

## Data Availability

The datasets generated during and/or analyzed during the current study are available from the corresponding author on reasonable request.
