# Supplementary material for: Oxidation-enhanced thermoelectric efficiency in a two-dimensional phosphorene oxide
Source: Sci Rep. 2021 Sep 17;11:18525. doi: 10.1038/s41598-021-97943-8 (PMC8448742; doi:10.1038/s41598-021-97943-8)
Supplement: Supplementary file 1 — Supplementary Information. [file 41598_2021_97943_MOESM1_ESM.pdf]

Supplementary Information

Oxidation-enhanced thermoelectric efficiency in a two-dimensional phosphorene oxide

Seungjun Lee[1], Jeong-Pil Song[1,2], Seoung-Hun Kang[1,3,*], and Young-Kyun Kwon[1,4,*]

[1]Department of Physics, Kyung Hee University, Seoul, 02447, Korea
[2]Department of Physics, University of Arizona, Tucson, Arizona, 85721, United States of America
[3]Materials Science and Technology Division, Oak Ridge National Laboratory, Oak Ridge, Tennessee, 37831, United States of America
[4]Department of Information Display and Research Institute for Basic Sciences, Kyung Hee University, Seoul, 02447, Korea
[*]Corresponding author: kangs@ornl.gov, ykkwon@khu.ac.kr

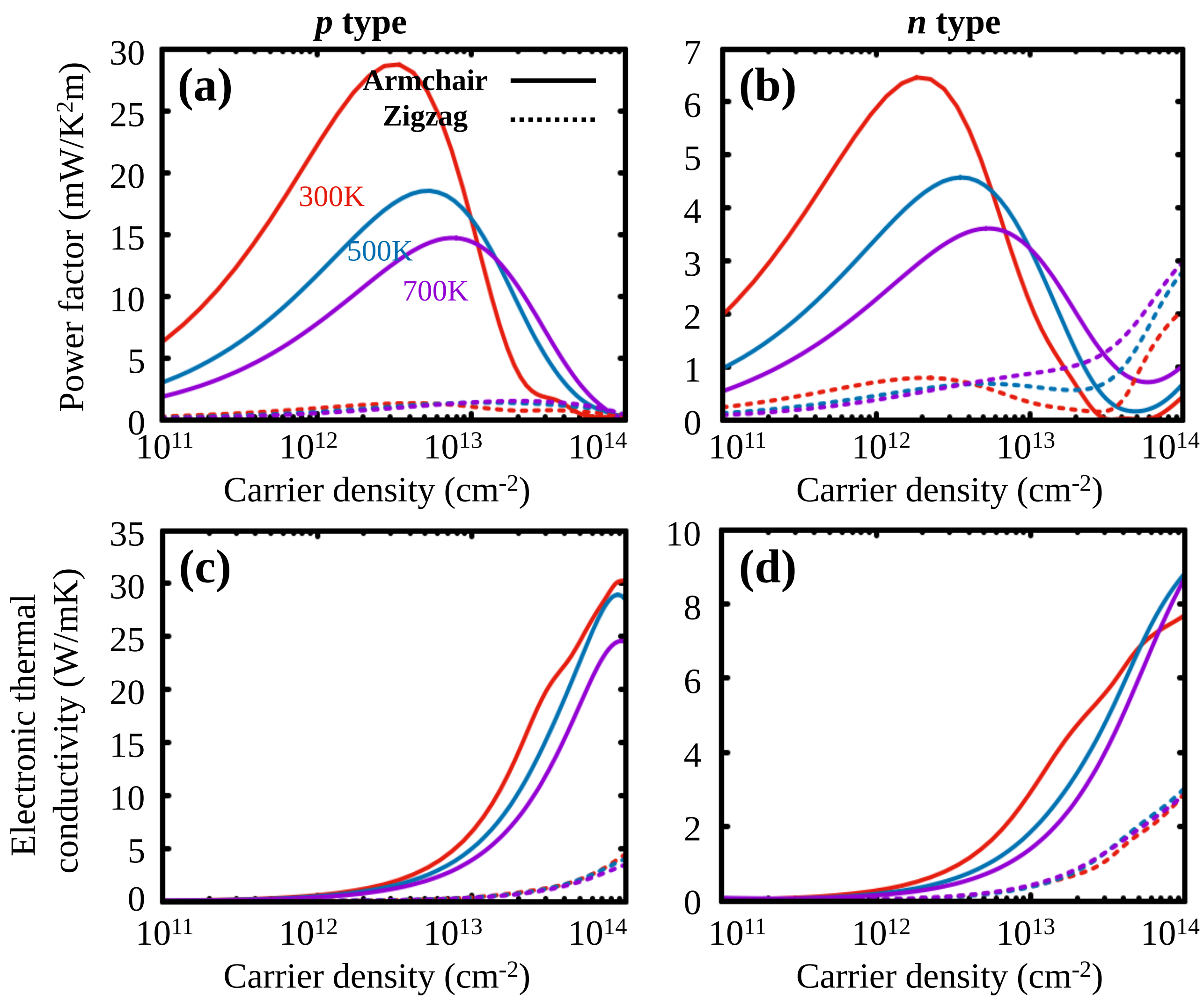

Figure S1. Power factors for (a) *p*-type and (b) *n*-type phosphorene and (c, d) their respective electronic thermal conductivities as a function of 2D carrier density. Solid and dashed lines denote these quantities computed along the armchair and zigzag transport directions at three different temperatures of 300, 500, and 700 K represented with the red, blue, and purple colors, respectively.

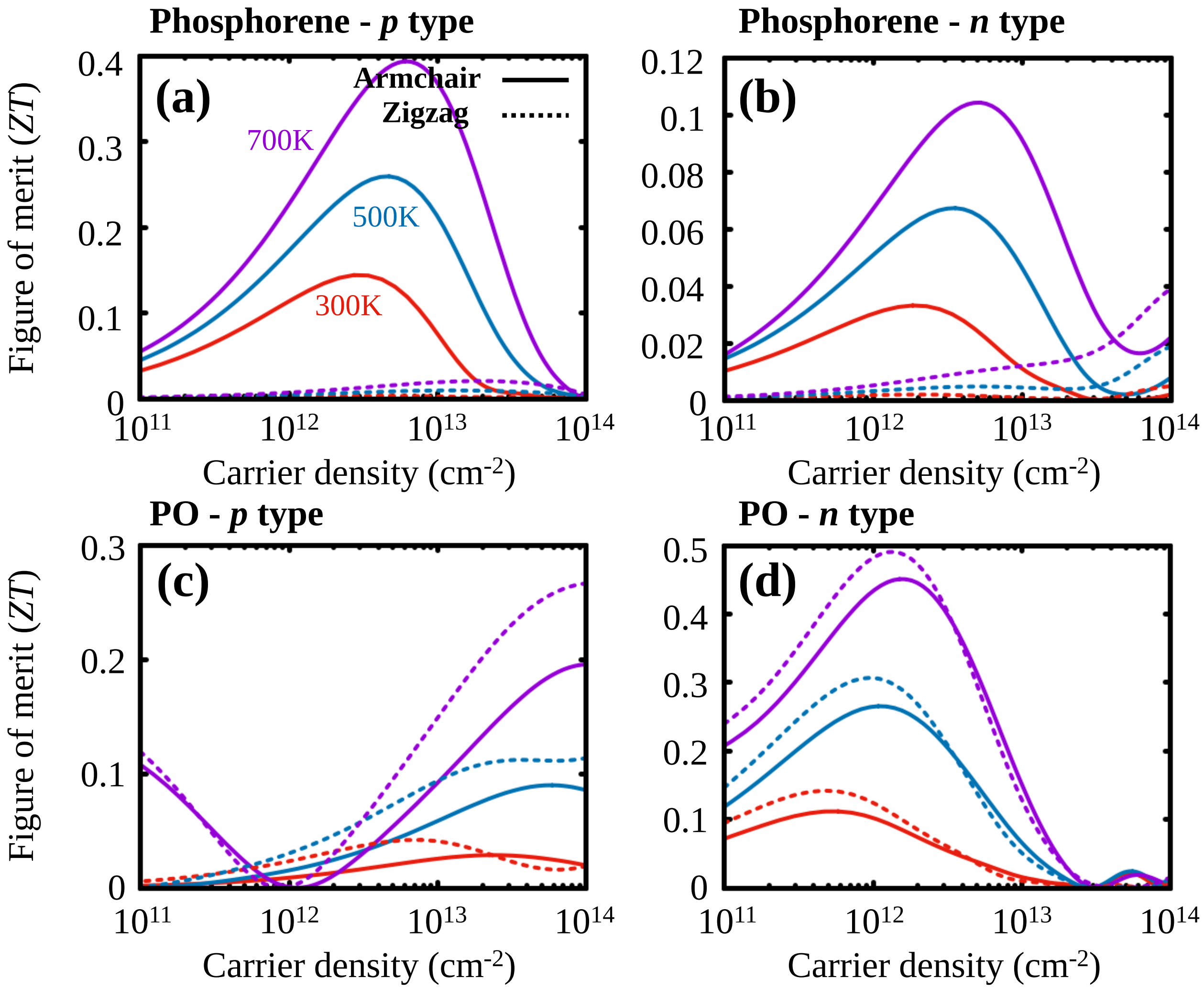

Figure S2. Thermoelectric figures of merit (ZT) of (a) p- and (b) n-doped phosphorene and (c, d) their PO counterparts as a function of 2D carrier density. Solid and dashed lines denote these quantities computed along the armchair and zigzag transport directions at three different temperatures of 300, 500, and 700 K represented with the red, blue, and purple colors, respectively.
